# Treatment patterns and burden of infection in patients with chronic lymphocytic leukemia and secondary immunodeficiency: a retrospective database study

**DOI:** 10.1007/s00277-024-05984-6

**Published:** 2024-09-12

**Authors:** Csaba Siffel, Joshua Richter, Colin Anderson-Smits, Marta Kamieniak, Kaili Ren, Drishti Shah, Matthew S. Davids

**Affiliations:** 1 Takeda Development Center Americas, Inc, Cambridge, MA USA; 2 College of Allied Health Sciences, Augusta University, Augusta, GA USA; 3 Tisch Cancer Institute, Icahn School of Medicine at Mount Sinai, New York, NY USA; 4 Department of Medical Oncology, Dana-Farber Cancer Institute, Boston, MA USA

**Keywords:** Chronic lymphocytic leukemia, Small lymphocytic lymphoma, Quality of life, Burden of illness

## Abstract

Patients with chronic lymphocytic leukemia/small lymphocytic lymphoma (CLL/SLL) and secondary immunodeficiency disease (SID) are susceptible to severe, recurrent, or persistent infections. This retrospective cohort study assessed the burden of infection in patients with CLL/SLL with and without SID, and in immunoglobulin replacement therapy (IgRT)-treated and -untreated patients with CLL/SLL and SID. Anonymized data from the US Optum-Humedica database (Oct-1-2015–Mar-10-2020) were used. Eligible patients aged ≥ 18 years with a confirmed CLL/SLL diagnosis were assigned to cohorts (SID or no-SID) using an algorithm based on serum IgG levels < 5.0 g/L, hypogammaglobulinemia diagnosis codes, and ≥ 1 major infection. A further sub-categorization was made based on patients with SID who received IgRT and those who did not. During 12-month follow-up, patients with CLL/SLL and SID were significantly more likely to experience infections (70.1% vs. 30.4%), including severe bacterial infections (39.8% vs. 9.2%), and infections requiring hospitalization (27.7% vs. 5.8%) than patients without SID. The use of anti-infectives and healthcare resource utilization (HCRU) was also higher in the SID cohort versus the no-SID cohort. Overall survival was shorter in patients with SID than those without (12.3 vs. 16.9 months). In patients with CLL/SLL and SID, burden of infection and HCRU were greater in IgRT-treated patients than in no-IgRT patients, potentially highlighting the IgRT-treated cohort as a more vulnerable population. Increasing understanding of SID burden may help to improve outcomes in patients with CLL/SLL. Further research is needed to develop guidance for IgRT use and to assess the benefits of IgRT in this vulnerable population.

**Supplementary Information:**

The online version contains supplementary material available at 10.1007/s00277-024-05984-6.

## Introduction

Patients with chronic lymphocytic leukemia (CLL)/small lymphocytic lymphoma (SLL) are at risk of developing secondary immunodeficiency disease (SID) owing to the underlying disease process and immunosuppressive anti-cancer treatment [1]. These patients are susceptible to severe, recurrent, or persistent infections that can impair patients’ quality of life and result in poor patient outcomes and a substantial clinical and economic burden [2, 3]. Furthermore, hypogammaglobulinemia has long been recognized as a risk factor for the development of this type of infection, with reports suggesting that up to 85% of all patients with CLL are also diagnosed with hypogammaglobulinemia [4, 5]. Poor outcomes in this patient population have been demonstrated in several studies. In a large Danish Cancer Register study, among 10,455 patients with CLL, 18.8–33.7% of deaths were infection related, compared with 13.8–22.3% of deaths in matched controls [6]. In CLL, prolonged exposure to antigen leads to an upregulation of T cell inhibitory receptors that become increasingly dysfunctional, which may contribute toward increased risk for infection [7]. In a cohort study in Spain, patients with monoclonal B cell lymphocytosis (*n* = 91) were shown to have increasing T cell and natural killer cell numbers in the peripheral blood over time, as well as a significantly shorter overall survival and significantly higher rate of infection as a cause of death compared with matched patients from the general population [8]. Another study assessing survival outcomes in patients with CLL, subsequently diagnosed with COVID-19, reported a case fatality rate of 33% (66/198 patients) at the time of data cut-off [9].

Although outcomes and prognosis for patients with mature B-cell malignancies and SID are poor, there is currently still limited guidance for helping clinicians to identify and treat patients at highest risk of developing SID and subsequent infections [1, 2, 10, 11]. Patients are often managed using serum immunoglobulin G (IgG) level monitoring, prophylactic immunoglobulin replacement therapy (IgRT) and/or antibiotics, and treatment with IgRT by indication [12]. Despite guidelines recommending IgRT only after antibiotic prophylaxis failure [13], there are concerns that IgRT is not being used appropriately, with patients being managed reactively with antibiotics and only being treated with IgRT when they are already very ill and experiencing multiple severe and/or recurrent infections. One retrospective study concluded that only 24% of patients with SID and mature B-cell malignancies who were prescribed IgRT actually met the European Medicines Agency’s recommendations for IgRT use in this patient population. Another study suggested that only 36% of patients with a mandatory indication for IgRT in patients with CLL and SID were prescribed treatment according to local guidelines [14, 15].

IgRT is the mainstay therapy for patients with primary immunodeficiency diseases (PIDs), and therefore the rationale for the use of IgRT in patients with SID is mainly based on clinical experience in the treatment of PIDs [16, 17]. Evidence for IgRT use in SID from large randomized controlled clinical trials, particularly those that report on infection and mortality outcomes, is lacking, and the studies that have been conducted were completed in the late 1980s and the 1990s [18–21]. Since this time, the complexity of SID has increased because the patient populations, therapeutic protocols, and treatment landscape have changed (particularly with the introduction of targeted therapies for CLL [22]), as have the infection outcomes assessed in primary studies. In the absence of recent randomized clinical trials reporting the use of IgRT and survival analysis in current patient populations with SID, there is a need to gather real-world evidence on infection-related outcomes, treatment pattens, and survival analysis in this population. Such evidence may aid clinical decision-making in the use of IgRT in patients with SID.

The aim of this study was to gather evidence on the burden of infection in patients with CLL/SLL. The demographics, clinical characteristics, numbers and types of infection, healthcare resource utilization (HCRU), treatment patterns, and overall survival in patients with SID compared with those without SID are described. Furthermore, these outcomes are compared between patients with SID who were treated with IgRT and those not treated with IgRT.

## Methods

### Study design

This retrospective, observational cohort study used anonymized data from the Optum-Humedica integrated claims and electronic health record database in the USA between October 1, 2015 and March 10, 2020 (Supplementary Fig. 1). The analysis described herein was one part of a broader analysis to assess outcomes in different B-cell malignancies (including multiple myeloma [MM] and non-Hodgkin lymphoma [NHL]) with or without SID; results of the combined analysis are reported elsewhere [23]. In the current analysis, diagnosis of CLL/SLL was defined as having at least two International Classification of Diseases, Tenth Revision, Clinical Modification (ICD-10-CM) diagnosis codes for CLL/SLL (C91.1X for CLL or C83.0x for SLL), occurring at least 30 days apart in a 6-month pre-index period (PIP). Patients with SID (SID cohort) and without SID (no-SID cohort) were identified from April 1, 2016 to March 10, 2019 (the selection window). In the SID cohort, the index date was the date of the first SID record, defined as the earliest occurrence of an ICD-10-CM code for hypogammaglobulinemia or a record of serum IgG levels < 5.0 g/L, depending on the operational definition of SID that was met. In the no-SID cohort, the index date was randomly assigned to replicate the distribution of index dates in the SID cohort.

Further stratification of patients with SID was performed according to whether IgRT was received. Patients with IgRT (IgRT-treated cohort) were re-indexed to the first IgRT treatment date after the SID index date. Those who did not receive IgRT (no-IgRT cohort) had a randomly assigned index date to replicate the distribution of index dates in the IgRT-treated cohort.

### Study population

Eligible patients were aged 18 years and older at the index date, with CLL/SLL clinical activity (defined as at least one medical or pharmacy claim of any type, as a proxy for continuous enrollment) for at least 6 months before and for at least 3 months after the index date (ending at the first occurrence of last alive date or end of study date). Full details of inclusion and exclusion criteria and the SID operational definition have been published previously [23]. In brief, patients in the SID cohort were defined as having either serum IgG levels < 5.0 g/L, specific antibody failure, ICD-10-CM codes for non-familial hypogammaglobulinemia (D80.1), selective IgG deficiency (D80.3), or antibody deficiency with near-normal immunoglobulins (D80.6), and the presence of at least one major infection during the selection window. Infections were defined as bacteremia or sepsis, bacterial meningitis, osteomyelitis/septic arthritis, bacterial pneumonia, visceral abscess, or opportunistic infections including listeriosis, fungal infections, viral infections including cytomegalovirus, hepatitis B virus, and hepatitis C virus. Patients in the no-SID cohort were defined as those who did not meet the inclusion criteria for the SID cohort (based on a predefined algorithm) but who met the criteria for CLL/SLL diagnosis during the PIP. Patients with PID during the PIP were excluded. Patients with evidence of SID during the PIP were also excluded to ensure inclusion of only patients with newly diagnosed SID.

After identification of patients with SID in the CLL/SLL population, a further sub-categorization was made based on those who received IgRT and those who did not. For IgRT/no-IgRT cohorts, eligible patients had clinical activity for at least 3 months after the IgRT/pseudo-index date (minimum 3-month and variable 12-month follow-up period) and for 6 months before the IgRT/pseudo-index date (in the PIP). For the IgRT-treated cohort, patients were required to have had repeated IgRT exposure (receipt of IgRT on two separate dates between the index date and the end of the study period). All patients who were not assigned an IgRT index date during the selection window were included in the no-IgRT cohort. Patients with IgRT use before the IgRT/pseudo-index date in the PIP were also excluded to ensure inclusion of only IgRT-naive patients.

### Outcomes

The following outcomes were assessed using data from patients with at least 12-month follow-up: baseline demographics (reported on the index date or as close to index as possible) and clinical characteristics (measured during the PIP, not including the index date); number, type, and severity of infections and bacterial infections; HCRU (infection-associated hospitalizations, all-cause inpatient admissions, outpatient services utilization, and pharmacy utilization); and treatment patterns (anti-infectives, anti-cancer treatments, and supportive care agents). Severe infections were defined as: failure to respond to oral antibiotics (defined as a switch in antibiotics or the addition of another antibiotic to the patient’s regimen), presence of at least one claim for an intravenous antibiotic, or presence of at least one claim for at least two oral antibiotics; hospitalization with any ICD-10-CM code for infection; infection with an unusual pathogen (*Clostridium difficile*, *Neisseria* sp., *Giardia* sp.); or unusual complications (mastoiditis, pleural effusion, abscess). Severe bacterial infections were defined using ICD-10-CM codes for bacteremia or sepsis, bacterial meningitis, osteomyelitis/septic arthritis, bacterial pneumonia, and visceral abscess. Overall survival was assessed in patients with at least 3 months of follow-up. The IgRT-treated and no-IgRT cohorts were also assessed for clinical and treatment-related outcomes, as available, among those patients with fixed 12-month follow-up data.

### Statistical analysis

Descriptive statistics were produced for all relevant study measures. Bivariate comparisons between the SID and no-SID cohorts were conducted using parametric independent sample *t*-tests (mean) and non-parametric Wilcoxon rank-sum tests (median) for continuous variables, and χ^2^ tests or Fisher’s exact tests for categorical variables. A *P* value of < 0.05 was considered statistically significant. Overall survival (time to death) was analyzed using the Kaplan–Meier method.

Similar statistical analyses were performed for comparison of the IgRT-treated and no-IgRT cohorts of patients with SID before matching. To adjust for observable selection bias and potentially confounding factors, patients in the IgRT-treated cohort were matched 1:1 to patients in the no-IgRT cohort via propensity score matching. Variables to be included in the match were determined after review of descriptive pre-match (unmatched) results, and comprised age, anti-CD20 monoclonal antibody use, duration between SID index date and treatment index date, duration of cancer, any infection and secondary/other malignancies. The propensity scores represent a patient’s probability of receiving a given treatment option and were calculated by summing coefficient values for potential confounding variables. Propensity scores were generated using a multivariable logistic regression model in which the dependent variable was IgRT use (yes/no). For matched cohorts, bivariate comparisons were conducted using parametric paired *t*-tests (mean) and non-parametric Wilcoxon signed-rank tests (median) for continuous variables and the McNemar’s test for categorical variables. Standardized mean differences (SMDs) were reported for the unmatched and matched cohorts as a measure of balance. SMD was calculated as the difference in means or proportions of a variable divided by the pooled standard deviation (SD). SMD of ≥ 0.10 between groups was considered meaningful and would indicate imbalance.

## Results

### Patient disposition

Of 4430 patients with CLL/SLL with at least 12 months of follow-up, 502 and 3928 patients were included in the SID and no-SID cohorts, respectively. Before matching, 142 and 267 of the patients in the SID cohort were included in the IgRT-treated and no-IgRT cohorts, respectively. Analyses of matched IgRT-treated and no-IgRT cohorts were performed using data from 118 patients from each cohort.

### Baseline demographics and clinical characteristics

Baseline demographics and clinical characteristics of the SID and no-SID cohorts are shown in Table 1. Compared with the no-SID cohort, the SID cohort had a higher mean (SD) Charlson Comorbidity Index score (3.5 [1.9] vs. 3.2 [1.7]; *P* = 0.003) and a longer duration of cancer (17.3 [10.0] vs. 15.4 [9.8] months; *P* < 0.001). Compared with the no-SID cohort, significantly more patients in the SID cohort had experienced an infection (56.0% vs. 22.6%) or severe bacterial infection (28.1% vs. 6.7%) and had greater exposure to anti-infectives (71.7% vs. 38.8% of patients); all *P* < 0.001.

**Table 1:** Baseline characteristics for SID and no-SID cohorts

	SID cohort (*n* = 502)	No-SID cohort (*n* = 3928)	*P* value
Demographic characteristics			
Age, years, mean (SD)	70.6 (9.7)	71.6 (10.5)	0.033
Age category, years, *n* (%)			0.041
18–34	0	5 (0.1)	
35–44	4 (0.8)	34 (0.9)	
45–54	22 (4.4)	189 (4.8)	
55–64	111 (22.1)	804 (20.5)	
65–74	183 (36.5)	1195 (30.4)	
≥ 75	182 (36.3)	1701 (43.3)	
Female, *n* (%)	205 (40.8)	1657 (42.2)	0.731
Race, *n* (%)			0.025
White/Caucasian	476 (94.8)	3566 (90.8)	
Black/African American	17 (3.4)	217 (5.5)	
Asian	1 (0.2)	21 (0.5)	
Other/unknown	8 (1.6)	124 (3.2)	
Ethnicity, *n* (%)			0.213
Hispanic/Latino	10 (2.0)	65 (1.7)	
Not Hispanic/Latino	477 (95.0)	3681 (93.7)	
Unknown	15 (3.0)	182 (4.6)	
Clinical characteristics			
Disease severity, *n* (%)			NR
In remission	34 (6.8)	187 (4.8)	
In relapse	9 (1.8)	53 (1.3)	
Not achieved remission	443 (88.2)	3439 (87.6)	
Missing/unknown	16 (3.2)	249 (6.3)	
Charlson Comorbidity Index score, mean (SD)^a^	3.5 (1.9)	3.2 (1.7)	0.003
Charlson Comorbidity Index score category, *n* (%)			0.002
0–2	211 (42.0)	1973 (50.2)	
3	120 (23.9)	819 (20.9)	
≥ 4	171 (34.1)	1136 (28.9)	
Duration of cancer, months, mean (SD)	17.3 (10.0)	15.4 (9.8)	< 0.001
Infection, *n* (%)			
Any	281 (56.0)	887 (22.6)	< 0.001
Severe bacterial infection	141 (28.1)	262 (6.7)	< 0.001
Exposed to immunosuppressants, *n* (%)	7 (1.4)	7 (0.2)	< 0.001
Exposed to anti-infectives, *n* (%)	360 (71.7)	1526 (38.8)	< 0.001
ECOG performance status, *n* (%)			0.037
0	51 (10.2)	402 (10.2)	
1	33 (6.6)	185 (4.7)	
2	5 (1.0)	30 (0.8)	
3	5 (1.0)	9 (0.2)	
Missing	408 (81.3)	3302 (84.1)	
Serum IgG level, g/L			
Mean (SD)	6.6 (2.5)	9.0 (3.4)	< 0.001
Patients with level < 5 g/L, *n* (%)	0	0	

^a^Comorbidities of interest were also examined and included congestive heart failure, coronary artery disease, chronic obstructive pulmonary disease, chronic renal disease, diabetes mellitus, hypertension, secondary/other malignancies, thyroid disease, rheumatologic disease, rheumatoid arthritis, cytopenia, idiopathic thrombocytopenia and renal insufficiency. The most common comorbidities of interest were hypertension, secondary/other malignancies and cytopenia in the SID cohort, and hypertension, secondary/other malignancies and diabetes mellitus in the no-SID cohort. *ECOG*, Eastern Cooperative Oncology Group; *IgG*, immunoglobulin G; *no-SID*, patients without secondary immunodeficiency disease; *NR*, not reported; *SD*, standard deviation; *SID*, patients with secondary immunodeficiency disease

Baseline demographics and clinical characteristics of the unmatched and matched IgRT-treated and no-IgRT cohorts are shown in Table 2. Before matching, imbalances between the cohorts were noted with regard to distribution in the categories for age group, Charlson Comorbidity Index score, infections, exposure to anti-infectives, and serum IgG levels < 5 g/L (all SMD > 10%). After matching, imbalances in distribution between the cohorts were still seen for Charlson Comorbidity Index score, severe bacterial infections, and exposure to anti-infectives, but also for race and ethnicity. In the matched cohorts, IgRT-treated patients had a numerically higher mean (SD) Charlson Comorbidity Index score than the no-IgRT matched cohort (3.8 [2.2] vs. 3.6 [1.9]; *P* = 0.346). Although similar proportions of patients in the matched cohorts had experienced any infection, numerically more patients in the IgRT-treated cohort than the no-IgRT cohort had experienced a severe bacterial infection at baseline (40.7% vs. 31.4%; *P* = 0.093) and a statistically significant greater proportion had previous exposure to anti-infectives (86.4% vs. 69.5%; *P* = 0.001). Furthermore, more patients in the IgRT-treated cohort than in the no-IgRT cohort had serum IgG levels < 5 g/L (72.0% vs. 54.2%; *P* = 0.007). Similar patterns in baseline characteristics were seen for the unmatched cohorts, except for the occurrence of any infection and severe bacterial infection, and exposure to anti-infectives, for which proportions were significantly higher in the IgRT-treated cohort compared with the no-IgRT cohort (all *P* < 0.001) (Table 2).

**Table 2:** Baseline characteristics for IgRT-treated and no-IgRT unmatched and matched cohorts of patients with SID

	Unmatched cohorts				Matched cohorts			
	IgRT-treated (*n* = 142)	No-IgRT (*n* = 267)	*P* value	SMD	IgRT-treated (*n* = 118)	No-IgRT (*n* = 118)	*P* value	SMD
Demographic characteristics								
Age, years, mean (SD)	70.0 (9.3)	71.1 (10.1)	0.279	−0.11	70.5 (9.3)	71.1 (10.0)	0.578	−0.06
Age category, years, *n* (%)			0.248	0.25			0.809	0.13
18–54	8 (5.6)	11 (4.1)			6 (5.1)	6 (5.1)		
55–64	32 (22.5)	59 (22.1)			26 (22.0)	20 (16.9)		
65–74	59 (41.5)	91 (34.1)			46 (39.0)	51 (43.2)		
≥ 75	43 (30.3)	106 (39.7)			40 (33.9)	41 (34.7)		
Gender, *n* (%)			0.206	−0.13			0.31	−0.14
Female	52 (36.6)	115 (43.1)			45 (38.1)	53 (44.9)		
Male	90 (63.4)	152 (56.9)			73 (61.9)	65 (55.1)		
Race, *n* (%)			0.813	0.08			0.392	0.09
White/Caucasian	135 (95.1)	251 (94.0)			111 (94.1)	109 (92.4)		
Black/African American	6 (4.2)	10 (3.7)			6 (5.1)	7 (5.9)		
Asian	0	1 (0.4)			0	0		
Other/unknown	1 (0.7)	5 (1.9)			1 (0.8)	2 (1.7)		
Ethnicity, *n* (%)			0.425	0.14			0.801	0.11
Hispanic/Latino	1 (0.7)	7 (2.6)			1 (0.8)	2 (1.7)		
Not Hispanic/Latino	137 (96.5)	250 (93.6)			115 (97.5)	112 (94.9)		
Unknown	4 (2.8)	10 (3.7)			2 (1.7)	4 (3.4)		
Clinical characteristics								
Disease severity, *n* (%)			NR	NR			NR	NR
In remission	9 (6.3)	18 (6.7)			8 (6.8)	8 (6.8)		
In relapse	5 (3.5)	6 (2.2)			3 (2.5)	2 (1.7)		
Remission not achieved	126 (88.7)	233 (87.3)			105 (89.0)	104 (88.1)		
Missing/unknown	2 (1.4)	10 (3.7)			2 (1.7)	4 (3.4)		
Charlson Comorbidity Index score, mean (SD)^a^	3.9 (2.2)	3.5 (1.9)	0.053	0.20	3.8 (2.2)	3.6 (1.9)	0.346	0.12
Charlson Comorbidity Index score category, *n* (%)			0.305	0.15			0.918	0.07
0–2	48 (33.8)	107 (40.1)			41 (34.7)	45 (38.1)		
3	34 (23.9)	67 (25.1)			30 (25.4)	27 (22.9)		
≥ 4	60 (42.3)	93 (34.8)			47 (39.8)	46 (39.0)		
Duration of cancer, months, mean (SD)	19.6 (10.0)	20.3 (11.3)	0.485	−0.07	20.4 (10.4)	20.3 (10.9)	0.924	0.01
Infection, *n* (%)								
Any	99 (69.7)	126 (47.2)	< 0.001	0.47	78 (66.1)	72 (61.0)	0.18	0.11
Severe bacterial infection	63 (44.4)	64 (24.0)	< 0.001	0.44	48 (40.7)	37 (31.4)	0.093	0.20
Exposed to immunosuppressants, *n* (%)	2 (1.4)	4 (1.5)	1.000	−0.01	1 (0.8)	3 (2.5)	0.317	−0.13
Exposed to anti-infectives, *n* (%)	126 (88.7)	174 (65.2)	< 0.001	0.58	102 (86.4)	82 (69.5)	0.001	0.42
ECOG performance status, *n* (%)			1.000	0.06			0.317	0.17
0–2	23 (16.2)	47 (17.6)			15 (12.7)	24 (20.3)		
3–4	2 (1.4)	5 (1.9)			1 (0.8)	3 (2.5)		
Missing	117 (82.4)	215 (80.5)			102 (86.4)	91 (77.1)		
Serum IgG level, g/L								
Mean (SD)	4.0 (3.7)	4.4 (1.8)	0.245	−0.14	4.1 (3.9)	4.4 (2.0)	0.651	−0.1
Patients with level < 5 g/L, *n* (%)	102 (71.8)	164 (61.4)	0.036	0.22	85 (72.0)	64 (54.2)	0.007	0.38

^a^Comorbidities of interest were also examined and included congestive heart failure, coronary artery disease, chronic obstructive pulmonary disease, chronic renal disease, diabetes mellitus, hypertension, secondary/other malignancies, thyroid disease, rheumatologic disease, rheumatoid arthritis, cytopenia, idiopathic thrombocytopenia and renal insufficiency. The most common comorbidities of interest were hypertension, secondary/other malignancies and cytopenia in the IgRT-treated cohort, and hypertension, secondary/other malignancies and coronary artery disease in the no-IgRT cohort. *ECOG*, Eastern Cooperative Oncology Group; *IgG*, immunoglobulin G; *IgRT*, immunoglobulin replacement therapy; *NA*, not available; *no-IgRT*, patients not treated with immunoglobulin replacement therapy; *NR*, not reported; *SD*, standard deviation; *SID*, secondary immunodeficiency disease; *SMD*, standardized mean difference

### Infections

At 12-month follow-up, a higher mean (SD) number of infections was reported in the SID cohort than in the no-SID cohort (8.4 [12.8] vs. 4.1 [5.4]; *P* < 0.001) and in the matched IgRT-treated cohort than in the no-IgRT cohort (10.9 [16.4] vs. 6.1 [6.9]; *P* = 0.002). A higher mean (SD) number of severe infections was also reported in the SID cohort than in the no-SID cohort (7.8 [9.5] vs. 4.6 [4.9]; *P* < 0.001) and in the matched IgRT-treated cohort than in the no-IgRT cohort (9.9 [10.8] vs. 4.3 [3.0]; *P* = 0.072).

**Fig. 1:**
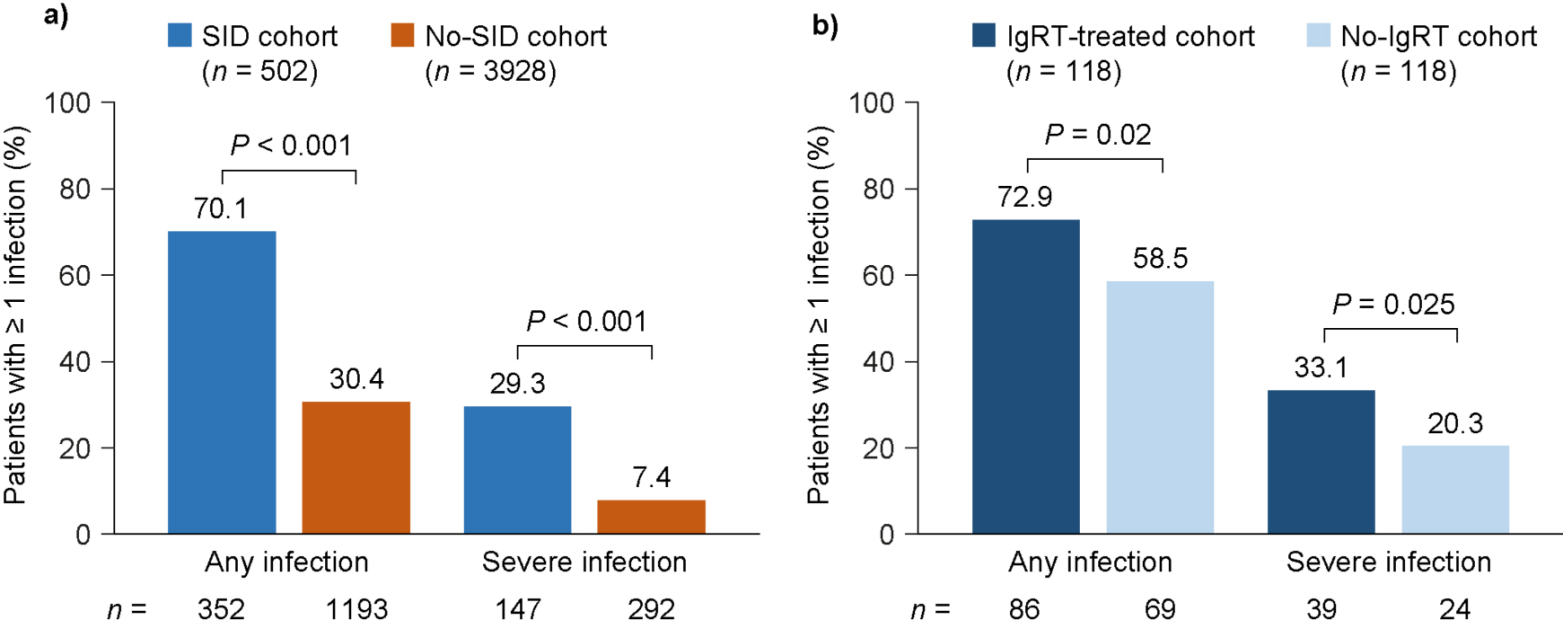
Occurrence of infections in the SID and no-SID cohorts (**a**) and in the matched IgRT-treated and no-IgRT cohorts of patients with SID (**b**), at 12-month follow-up. *IgRT*, immunoglobulin replacement therapy; *no-IgRT*, patients not treated with immunoglobulin replacement therapy; *no-SID*, patients without secondary immunodeficiency disease; *SID*, secondary immunodeficiency disease

**Fig. 2:**
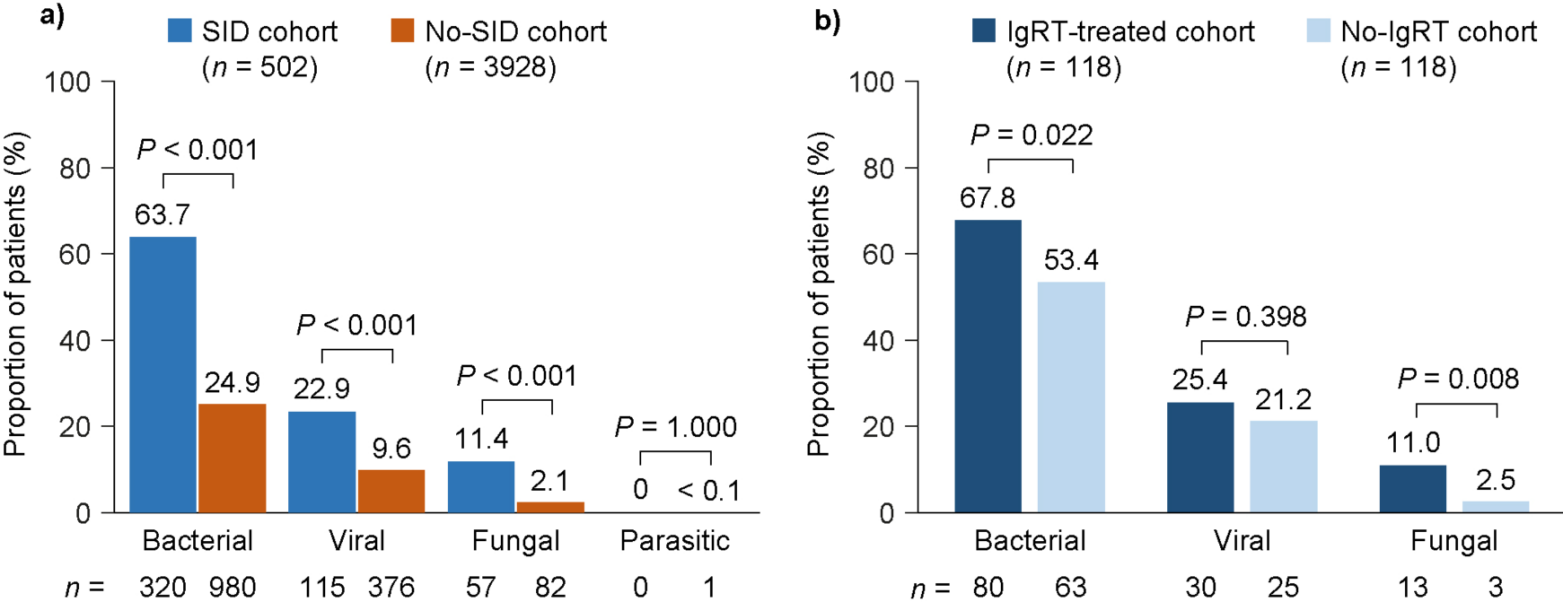
Types of infection in the SID and no-SID cohorts (**a**) and in the matched IgRT-treated and no-IgRT cohorts of patients with SID (**b**), at 12-month follow-up. *IgRT*, immunoglobulin replacement therapy; *no-IgRT*, patients not treated with immunoglobulin replacement therapy; *no-SID*, patients without secondary immunodeficiency disease; *SID*, secondary immunodeficiency disease

The numbers of patients experiencing at least one infection (any or severe) are shown in Fig. 1. The most common type of infection in all cohorts was bacterial (Fig. 2).

A higher mean (SD) number of bacterial infections was reported in the SID cohort than in the no-SID cohort (7.6 [10.4] vs. 4.1 [5.4]; *P* < 0.001) and in the matched IgRT-treated cohort than in the no-IgRT cohort (9.2 [11.0] vs. 5.8 [6.6]; *P* = 0.005). Similarly, more patients in the SID cohort than the no-SID cohort experienced at least one severe bacterial infection (200 [39.8%] and 361 [9.2%], respectively; *P* < 0.001). The most frequently reported severe bacterial infections in each cohort were bacterial pneumonia (171 [34.1%] and 263 [6.7%], respectively; *P* < 0.001) and bacteremia/sepsis (88 [17.5%] and 126 [3.2%], respectively; *P* < 0.001). In the matched IgRT-treated and no-IgRT cohorts, a similar pattern was observed: 52 patients (44.1%) and 31 patients (26.3%), respectively, experienced at least one severe bacterial infection (*P* = 0.002), and the most frequently reported severe bacterial infections were bacterial pneumonia (45 [38.1%] and 27 [22.9%] in each cohort, respectively; *P* = 0.008) and bacteremia/sepsis (26 [22.0%] and 12 [10.2%] in each cohort, respectively; *P* = 0.004).

### HCRU

HCRU outcomes are summarized for the SID and no-SID cohorts in Table 3a and for the IgRT-treated and no-IgRT cohorts in Table 4a. Patients in the SID cohort were more likely to have had at least one all-cause hospitalization than patients in the no-SID cohort (38.4% vs. 13.3%; *P* < 0.001); this was also the case for the IgRT-treated cohort versus the no-IgRT cohort (39.0% vs. 24.6%; *P* = 0.015). The mean (SD) length of hospitalization was numerically longer in the SID cohort than in the no-SID cohort (7.8 [8.5] vs. 6.6 [17.9] days; *P* = 0.228) and also in the IgRT-treated cohort than in the no-IgRT cohort (8.9 [10.1] vs. 5.9 [2.8] days; *P* = 0.501). A greater proportion of patients experienced at least one infection-associated hospitalization in the SID cohort compared with the no-SID cohort (27.7% vs. 5.8%; *P* < 0.001) and in the IgRT-treated cohort compared with the no-IgRT cohort (29.7% vs. 19.5%; *P* < 0.064. Mean (SD) prescription fills were also higher in the SID cohort than in the no-SID cohort (24.9 [23.5] vs. 13.3 [13.1]; *P* < 0.001) and in the IgRT-treated cohort than in the no-IgRT cohort (32.0 [27.5] vs. 19.8 [18.8]; *P* < 0.001).

**Table 3:** HCRU (a) and treatment patterns (b) for SID and no-SID cohorts at 12-month follow-up

	SID cohort (*n* = 502)	No-SID cohort (*n* = 3928)	*P* value
(a) HCRU			
Patients with ≥ 1 of the listed healthcare resources, *n* (%)^a^			
Inpatient all-cause hospitalizations	193 (38.4)	524 (13.3)	< 0.001
Number per patient, mean (SD)	1.9 (1.3)	1.5 (1.1)	< 0.001
LOS, days, mean (SD)	7.8 (8.5)	6.6 (17.9)	0.228
Infection-associated hospitalizations	139 (27.7)	227 (5.8)	< 0.001
Number per patient, mean (SD)	8.0 (9.6)	5.3 (5.0)	0.002
LOS, days, mean (SD)	9.5 (13.1)	7.1 (7.7)	0.052
ER visit	194 (38.6)	822 (20.9)	< 0.001
Number per patient, mean (SD)	2.1 (2.0)	1.9 (1.5)	0.158
Physician office visit	448 (89.2)	3265 (83.1)	< 0.001
Number per patient, mean (SD)	16.7 (14.3)	10.9 (10.2)	< 0.001
Laboratory/pathology visit	501 (99.8)	3770 (96.0)	< 0.001
Number per patient, mean (SD)	24.0 (22.1)	11.1 (11.5)	< 0.001
Surgical service visit	32 (6.4)	116 (3.0)	< 0.001
Number per patient, mean (SD)	1.3 (0.6)	1.3 (0.6)	0.568
Radiology service visit	18 (3.6)	46 (1.2)	< 0.001
Number per patient, mean (SD)	1.2 (0.4)	1.7 (1.3)	0.027
Prescription fill	492 (98.0)	3420 (87.1)	< 0.001
Number per patient, mean (SD)	24.9 (23.5)	13.3 (13.1)	< 0.001
(b) Treatment patterns			
Patients with any use of the listed treatments, *n* (%)^a^			
Any anti-infective use	429 (85.5)	1908 (48.6)	< 0.001
Any antibiotic use	417 (83.1)	1791 (45.6)	< 0.001
Number of claims, mean (SD)	7.6 (11.6)	3.6 (4.8)	< 0.001
IV antibiotic use	234 (46.6)	660 (16.8)	< 0.001
Number of claims, mean (SD)	7.5 (11.7)	4.1 (5.3)	< 0.001
Any antiviral use	145 (28.9)	373 (9.5)	< 0.001
Number of claims, mean (SD)	6.0 (11.6)	2.9 (4.6)	0.003
IV antiviral use	2 (0.4)	2 (< 0.1)	0.066
Number of claims, mean (SD)	1.5 (0.7)	4.5 (3.5)	0.360
Any antifungal use	68 (13.5)	117 (3.0)	< 0.001
Number of claims, mean (SD)	6.5 (14.3)	2.5 (3.9)	0.027
IV antifungal use	19 (3.8)	10 (0.3)	< 0.001
Number of claims, mean (SD)	4.8 (4.3)	2.9 (3.7)	0.238
Any chemotherapeutic agent use	222 (44.2)	932 (23.7)	< 0.001
Number of claims, mean (SD)	7.2 (6.8)	5.3 (5.4)	< 0.001
Any use of supportive care agent	1 (0.2)	1 (< 0.1)	0.214
Number of claims, mean (SD)	3.0 (NE)	7.0 (NE)	NE

Mean (SD) number of each healthcare resource type per patient was calculated among patients with any use of that resource type. Mean (SD) number of claims for each treatment type was calculated among patients with any use of that treatment type. ^a^Unless otherwise stated. *ER*, emergency room; *HCRU*, healthcare resource utilization; *IV*, intravenous; *LOS*, length of stay; *NE*, not estimable; *no-SID*, patients without secondary immunodeficiency disease; *SD*, standard deviation; *SID*, patients with secondary immunodeficiency disease

**Table 4:** HCRU (a) and treatment patterns (b) for IgRT-treated and no-IgRT matched cohorts of patients with SID at 12-month follow-up

	IgRT-treated (*n* = 118)	No-IgRT (*n* = 118)	*P* value
(a) HCRU			
Patients with ≥ 1 of the listed healthcare resources, *n* (%)^a^			
Inpatient all-cause hospitalizations	46 (39.0)	29 (24.6)	0.015
Number per patient, mean (SD)	2.2 (1.8)	1.9 (1.1)	0.406
LOS, days, mean (SD)	8.9 (10.1)	5.9 (2.8)	0.501
Infection-associated hospitalizations	35 (29.7)	23 (19.5)	0.064
Number per patient, mean (SD)	10.9 (11.0)	4.3 (3.0)	0.066
LOS, days, mean (SD)	12.3 (20.6)	7.2 (3.7)	0.624
ER visit	44 (37.3)	30 (25.4)	0.048
Number per patient, mean (SD)	2.6 (3.1)	2.2 (2.0)	0.351
Physician office visit	111 (94.1)	102 (86.4)	0.050
Number per patient, mean (SD)	19.5 (17.3)	15.4 (12.3)	0.069
Laboratory/pathology visit	118 (100.0)	117 (99.2)	NA
Number per patient, mean (SD)	30.3 (26.8)	18.3 (16.7)	< 0.001
Surgical service visit	8 (6.8)	6 (5.1)	0.593
Number per patient, mean (SD)	1.5 (1.1)	1.0 (0.0)	NA
Radiology service visit	6 (5.1)	5 (4.2)	0.763
Number per patient, mean (SD)	1.2 (0.4)	1.2 (0.5)	NA
Prescription fill	117 (99.2)	113 (95.3)	0.102
Number per patient, mean (SD)	32.0 (27.5)	19.8 (18.8)	< 0.001
(b) Treatment patterns			
Patients with any use of the listed treatments, *n* (%)^a^			
Any anti-infective use	98 (83.1)	91 (77.1)	0.223
Any antibiotic use	95 (80.5)	87 (73.7)	0.206
Number of claims, mean (SD)	9.9 (12.2)	5.3 (5.8)	< 0.001
IV antibiotic use	58 (49.2)	40 (33.9)	0.018
Number of claims, mean (SD)	9.3 (10.9)	5.1 (6.3)	0.020
Any antiviral use	43 (36.4)	25 (21.2)	0.009
Number of claims, mean (SD)	8.4 (13.5)	2.5 (3.1)	1
IV antiviral use	0	1 (0.8)	NA
Number of claims, mean (SD)	0	1.0 (0.0)	NA
Any antifungal use	18 (15.3)	12 (10.2)	0.18
Number of claims, mean (SD)	7.0 (9.4)	3.8 (3.6)	0.643
IV antifungal use	3 (2.5)	2 (1.7)	0.655
Number of claims, mean (SD)	9.0 (5.3)	5.5 (0.7)	NA
Any chemotherapeutic agent use	56 (47.5)	49 (41.5)	0.385
Number of claims, mean (SD)	6.9 (6.5)	6.8 (5.8)	0.48

Mean (SD) number of each healthcare resource type per patient was calculated among patients with any use of that resource type. Mean (SD) number of claims for each treatment type was calculated among patients with any use of that treatment type. ^a^Unless otherwise stated. *ER*, emergency room; *IgRT*, immunoglobulin replacement therapy; *IV*, intravenous; *LOS*, length of stay; *NA*, not available; *NE*, not estimable; *no-IgRT*, patients not treated with immunoglobulin replacement therapy; *SD*, standard deviation; *SID*, secondary immunodeficiency disease

### Treatment patterns

Treatment patterns are summarized for the SID and no-SID cohorts in Table 3b and for the IgRT-treated and no-IgRT cohorts in Table 4b. Patients with SID were more likely to have had at least one claim for an anti-infective compared with patients without SID (85.5% vs. 48.6%; *P* < 0.001), the most common type in both cohorts being antibiotics (SID: 83.1%; no-SID: 45.6%). A similar pattern of anti-infective use was seen for the IgRT-treated versus no-IgRT cohorts (83.1% vs. 77.1%; *P =* 0.223), with antibiotics being the most common type of anti-infective used (80.5% vs. 73.7%; *P* = 0.206). For the IgRT-treated cohort, the median duration of IgRT was 311.5 days and there was a median of seven IgRT prescriptions filled per patient.

### Overall survival

**Fig. 3:**
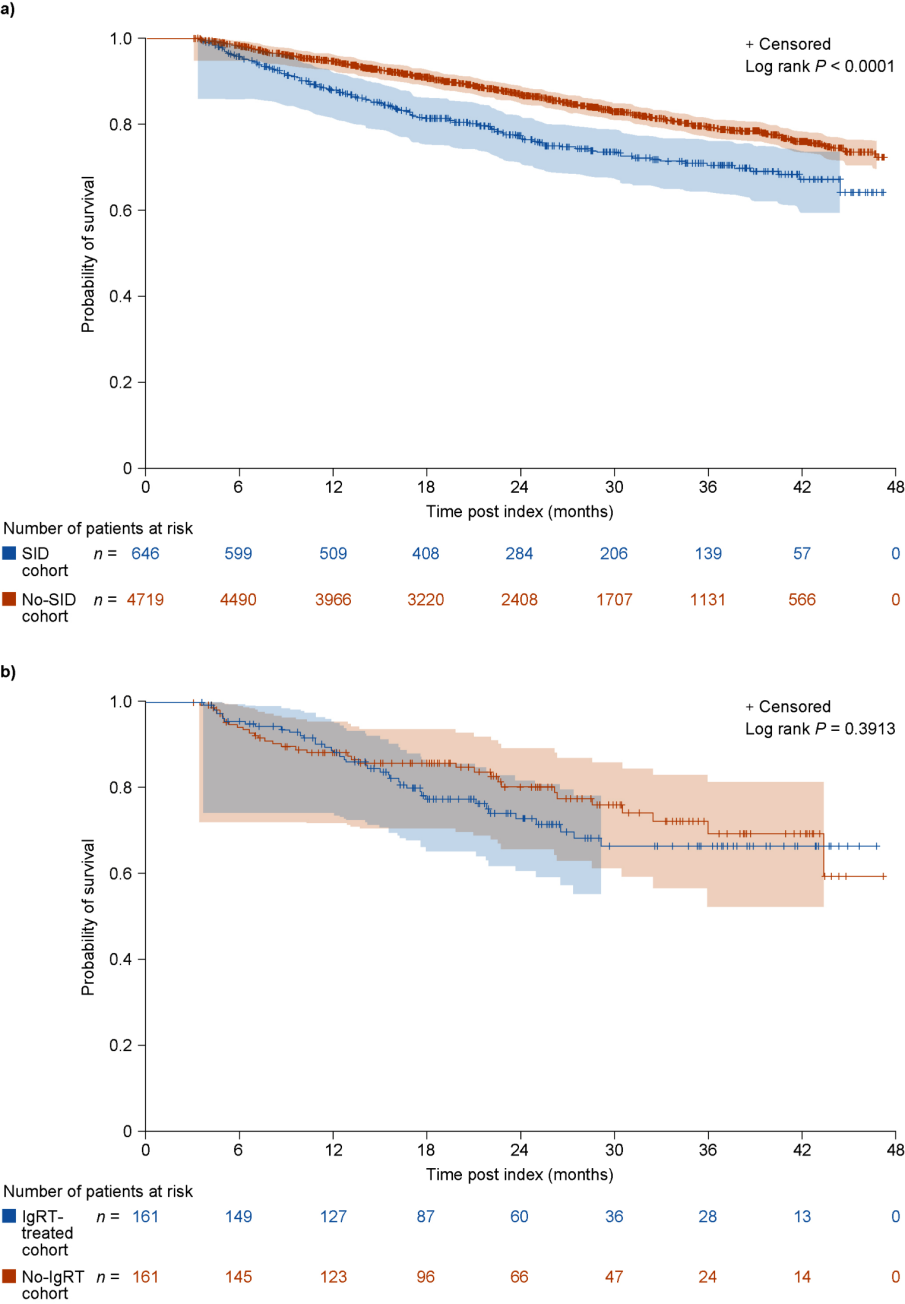
Kaplan–Meier curves showing overall survival in the SID and no-SID cohorts (**a**) and in the IgRT-treated and no-IgRT cohorts of patients with SID (**b**). Number of patients at risk and 95% Hall–Wellner bands are shown. *IgRT*, immunoglobulin replacement therapy; *no-IgRT*, patients not treated with immunoglobulin replacement therapy; *no-SID*, patients without secondary immunodeficiency disease; *SID*, secondary immunodeficiency disease

In those patients with a minimum of 3 months’ follow-up, there were 152 deaths (23.5%) in patients with SID and 714 deaths (15.1%) in those without SID. In patients who died, the median time to death was 12.3 and 16.9 months for patients in the SID and no-SID cohorts, respectively. Overall survival was shorter in the SID cohort than in the no-SID cohort (*P* < 0.001) (Fig. 3a), and this trend was observed at all time points (probability of survival [95% confidence intervals] at 6 months: 95.8% [93.9–97.1%] vs. 98.3% [97.9–98.6%]; 12 months: 88.0% [85.2–90.3%] vs. 94.7% [94.0–95.3%]; and 24 months: 77.3% [73.5–80.6%] vs. 87.2% [86.1–88.2%]).

In the IgRT-treated and no-IgRT cohorts, there were 40 deaths (24.8%) and 33 deaths (20.5%), respectively, with a median time to death of 12.8 and 9.6 months. Although probability of survival appeared to be lower in IgRT-treated patients than in no-IgRT patients, 95% confidence intervals overlapped at 6, 12, and 24 months, and the overall survival was comparable between patients with SID in the IgRT-treated and no-IgRT cohorts (*P* = 0.391) (Fig. 3b).

### Clinical and treatment response outcomes by IgRT cohort

Clinical and treatment response outcomes in the IgRT-treated and no-IgRT cohorts of patients with SID are seen in Table 5. The mean rate of infections during the 12-month follow-up period was 2.2-times higher in the matched IgRT-treated cohort compared with the matched no-IgRT cohort (mean, 7.9 vs. 3.6, respectively). Between-cohort differences were smaller for the incidences of hospitalizations and severe infections; nonetheless, incidences across all outcomes measured were higher in the IgRT-treated cohort than the no-IgRT cohort. Among the patients with available data, fewer patients in the IgRT-treated cohort than the no-IgRT cohort maintained an IgG level of < 5 g/L post-index and throughout the 12-month follow-up period; 75.4% of IgRT-treated patients had an increase in IgG serum levels to ≥ 5 g/L over the follow-up period compared with 17.8% of the no-IgRT cohort.

**Table 5:** Clinical and treatment response outcomes for IgRT-treated and no-IgRT matched cohorts of patients with SID at 12-month follow-up

	IgRT-treated (*n* = 118)	No-IgRT (*n* = 118)	Difference^a^	IRR^b^
Any infections				
Number per patient, mean (SD)	7.93 (14.80)	3.56 (6.06)		
Incidence	7.93	3.56	−4.37	2.23
Severe infections				
Number per patient, mean (SD)	3.26 (7.74)	0.86 (2.18)		
Incidence	3.26	0.86	−2.40	3.79
Anti-infective use				
Number per patient, mean (SD)	12.08 (20.98)	4.81 (7.05)		
Incidence	12.08	4.81	−7.27	2.51
Hospitalizations				
Number per patient, mean (SD)	0.84 (1.54)	0.47 (1.00)		
Incidence	0.84	0.47	−0.37	1.79
Length of hospital stay				
Mean (SD), days	8.41 (21.89)	2.73 (6.15)		
Serum IgG levels, *n* (%)				
Maintained level of < 5 g/L^c^	11 (9.3)	40 (33.9)		
Increased level to ≥ 5 g/L	89 (75.4)	21 (17.8)		
Missing data	18 (15.3)	57 (48.3)		

^a^Measured as the difference between the two cohorts: IgRT-treated − no-IgRT; ^b^measured as IgRT-treated ÷ no-IgRT; ^c^serum IgG level recorded at index date. *IgG*, immunoglobulin G; *IgRT*, immunoglobulin replacement therapy; *IRR*, incidence rate ratio; *no-IgRT*, patients not treated with immunoglobulin replacement therapy; *SD*, standard deviation; *SID*, secondary immunodeficiency disease

## Discussion

This study provides evidence that SID is a significant burden for patients with CLL/SLL, leaving this population vulnerable to infection and infectious complications. During the 12-month follow-up period, patients with CLL/SLL and SID were more likely to experience infections, including severe bacterial infections, and infections requiring hospitalization than patients without SID. In addition, the use of anti-infectives and HCRU was higher in the SID cohort versus the no-SID cohort. Overall survival was shorter in patients with SID than in those without SID. Furthermore, in those patients with CLL/SLL and SID, burden of infection and HCRU were greater in patients who had received IgRT than in those who had not received IgRT, which is potentially indicative of the IgRT-treated cohort being a more vulnerable population.

In the current study, patients with SID had shorter all-cause survival than those without SID: median time to death was 12.3 months in the SID cohort and 16.9 months in the no-SID cohort. In the SID cohort, the mean (SD) number of infections (any type) reported at 12-month follow-up was 8.4 (12.8), and for the number of severe infections it was 7.8 (9.5). In a US claims study using the IQVIA PharMetrics^®^ Plus database, after SID diagnosis, in patients with CLL the annualized mean (SD) number of any infections was 13.1 (28.7) and the annualized mean (SD) number of severe infections was 0.9 (2.6) [24]. When considering the infection rates reported in the current study, it is important to understand that each infection may have been counted more than once if it was recorded under the same diagnostic code for more than 1 day; this was most likely for severe infections that may have appeared in the data for several days. This potential source of bias might be addressed by implementing a requirement for a gap of 5 or 7 days, for example, between infections under the same diagnostic code to be recorded as separate instances. In the current study, 28% of patients in the SID cohort experienced at least one infection-associated hospitalization, and length of stay for an infection-associated hospitalization was 9.5 days. This rate of infection-associated hospitalizations is comparable with that reported in a previous study in which 39% of the 84 patients with CLL had at least one infection-associated hospitalization in the 12 months before enrollment [14].

The potential benefit of IgRT in patients with SID subsequent to mature B-cell malignancy is under-recognized, despite its potential to improve patient outcomes. This under-recognition is probably compounded by the lack of recent, good-quality evidence in support of IgRT and the difficulty in drawing robust conclusions from the available data owing to the lack of study uniformity [22]. This could potentially be addressed in future studies examining prophylactic use of IgRT in patients with SID. Although IgRT is not currently approved for prophylactic use in patients with SID to prevent infections, the potential benefits have been demonstrated for patients with hematological malignancy and SID in small studies [20, 25]. In patients with CLL/SLL and SID in the current study, the mean (SD) number of infections reported at 12-month follow-up was higher in the IgRT-treated cohort (10.9 [16.4]) than in the no-IgRT cohort (6.1 [6.9]), and a greater percentage of patients in the IgRT-treated cohort than in the no-IgRT cohort experienced at least one severe infection (33% and 20%, respectively). Furthermore, 30% of patients in the IgRT-treated cohort experienced at least one infection-associated hospitalization, compared with 19% of patients in the no-IgRT cohort. Clinical and treatment response outcomes in the IgRT-treated and no-IgRT cohorts followed similar trends (i.e., across all outcomes measured at 12-month follow-up, values were higher in the IgRT-treated cohort than in the no-IgRT cohort). In comparison, a previously published study that utilized the IQVIA PharMetrics Plus database reported a lower mean (SD) number of infections in an IgRT-treated SID cohort (5.8 [15.7]), and fewer patients (21%) experienced at least one severe bacterial infection in the 12 months after SID diagnosis [26]. It is noteworthy that despite the benefits reported in some studies [18–20], IgRT does not entirely overcome the risks of SID itself, but only partially so [21]. Therefore, it is probable that infections had already occurred in our study population for the treatment indication to have been made. In the current study, data were not available for the number of IgRT infusions received or the dose; there is a possibility that the number of infusions received was not sufficient and the dose sub-optimal. In a previous randomized trial of prophylactic IgRT for patients with CLL and SID and recurrent infections, IgRT reduced the number of infections compared with placebo, but there were treatment failures in 29% of patients [20]. These patients were then switched to an increased dose of IgRT (from 18 g to 24 g), with 57% remaining infection-free during the ensuing 1–3-month follow-up period [20]. This highlights the uncertainty around optimal IgRT dosing in SID subsequent to mature B-cell malignancy to achieve protective serum IgG levels. In the current study, the mean serum IgG level pre-index was lower in the IgRT-treated cohort than in the no-IgRT cohort, and a greater proportion of patients had levels < 5 g/L which is consistent with evidence that patients with CLL and SID are often started on IgRT once they are already in a condition of poor health [14, 15]. These data could also be interpreted as patients at baseline requiring IgRT in order to reach the same serum IgG level as patients not receiving IgRT, or alternatively, that sub-optimal dosing and infrequent administration of IgRT meant that treated patients were unable to reach the same IgG level as the no-IgRT group at baseline. However, at 12-month follow-up, a higher proportion of patients in the IgRT-treated cohort, compared with the no-IgRT cohort, experienced an increase in serum IgG levels to ≥ 5 g/L (relative to index levels).

The IgRT-treated cohort had a longer median survival time than the overall SID cohort (12.8 months vs. 12.3 months), while patients with SID not treated with IgRT had a shorter survival time than the overall SID cohort (9.6 months vs. 12.3 months). Overall survival was comparable between patients with SID in the IgRT-treated and no-IgRT cohorts, although probability of survival appeared to be lower in IgRT-treated patients than in no-IgRT patients at 24 months. It is possible that this observation is a result of the IgRT-treated patients having more heavily pretreated CLL than the patients who had not received IgRT, thereby leading to increased mortality due to CLL disease progression rather than infection.

A key strength of this study was the use of data from an electronic health record database rather than claims data. This ensured more robust criteria for SID, using an algorithm to identify patients based on the earliest occurrence of a diagnosis code indicating SID or a record of IgG serum level < 5.0 g/L, resulting in a lower risk of misclassification compared with previous studies. A limitation of this study was the retrospective database study design, including potential selection bias (although parameters were included to account for this) and the fact that there were no controls. There was also the potential for miscoding/misclassification owing to the lack of clinical detail with electronic medical records. Therefore, data were missing for some variables and disease severity was not well described, with many patients having unspecified disease severity. Guidance for evaluation, identification, and management of SID remains relatively limited. Other potential risk factors related to infection, such as T cell dysfunction, were not accounted for in this analysis. Furthermore, for the analysis of IgRT-treated and no-IgRT cohorts, matching was performed for a subgroup of patients with CLL as part of the larger study, of which this analysis of the CLL/SLL subgroup specifically is a part. Outcomes presented herein extracted results for the CLL/SLL patient subgroup from the combined matched group. Thus, although it is possible to build a general understanding of the effects of receiving or not receiving IgRT in patients with CLL/SLL and SID, there may be malignancy-specific nuances that were not captured. Comorbidities can be associated with infection related mortality in hematological malignancies [27, 28]. We found on the basis of Charlson Comorbidity Index scores that the baseline comorbidity profile was similar between the IgRT-treated and no-IgRT cohorts, both before and after matching; baseline Charlson Comorbidity Index score ≥ 3 was recorded for 69.9% vs. 67.9%, in the IgRT-treated and no-IgRT pre-matched cohorts and 65.3% vs. 61.9% in the post-matched cohorts, respectively.

It is clear that SID is a significant burden for patients with CLL/SLL, leaving them vulnerable to infection and infectious complications, which may include hospitalization and even death; therefore, better diagnostic and therapeutic guidelines and tools are required to identify patients with CLL/SLL who may be at high risk of developing SID. In addition, increasing the understanding of the SID burden may help to improve outcomes in this population and to define the standards for evaluation and management of SID. Although there is the potential for treatment with IgRT to improve outcomes in patients with CLL/SLL and SID, results from the present study suggest that the IgRT-treated cohort is a more vulnerable population of patients with SID subsequent to CLL/SLL than those for whom IgRT is not indicated. Further research, including prospective trials, is needed to develop guidance for IgRT use and to assess the benefits of IgRT in this vulnerable population.

## Electronic supplementary material

Below is the link to the electronic supplementary material.

Supplementary Material 1

## Data Availability

The data that support the findings of this study are available within the Humedica database. These data are not publicly available, but data are available from the corresponding author upon reasonable request and with the permission of Humedica.
