# Supplementary material for: Treatment patterns and burden of infection in patients with chronic lymphocytic leukemia and secondary immunodeficiency: a retrospective database study
Source: Ann Hematol. 2024 Sep 12;103(11):4567–80. doi: 10.1007/s00277-024-05984-6 (PMC11534992; doi:10.1007/s00277-024-05984-6)

**Treatment patterns and burden of infection in patients with chronic lymphocytic leukemia and secondary immunodeficiency: a retrospective database study**

*Annals of Hematology*

Csaba Siffel^1,2^ • Joshua Richter • Colin Anderson-Smits • Marta Kamieniak • Kaili Ren • Drishti Shah • Matthew S. Davids

^1^ Takeda Development Center Americas, Inc., Cambridge, MA, USA

^2^ College of Allied Health Sciences, Augusta University, Augusta, GA, USA

Corresponding author: Csaba Siffel (csaba.siffel@takeda.com)

# Supplementary information

**Supplementary Fig. 1** Study design. *CLL*, chronic lymphocytic leukemia; *ICD-10-CM*, International Classification of Diseases, Tenth Revision, Clinical Modification; *IgG*, immunoglobulin G; *IgRT*, immunoglobulin replacement therapy; *no-IgRT*, patients not treated with immunoglobulin replacement therapy; *no-SID*, patients without secondary immunodeficiency disease; *SID*, secondary immunodeficiency disease; *SLL*, small lymphocytic lymphoma


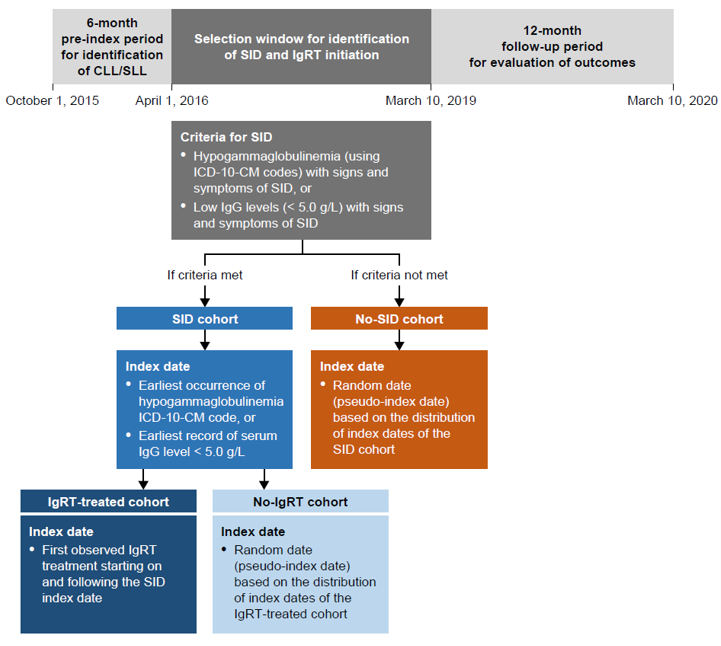

Supplement: Supplementary file 1 — Supplementary Material 1 [file 277_2024_5984_MOESM1_ESM.docx]
